# The role of hepatocyte nuclear factor 4alpha in metastatic tumor formation of hepatocellular carcinoma and its close relationship with the mesenchymal–epithelial transition markers

**DOI:** 10.1186/1471-2407-13-432

**Published:** 2013-09-23

**Authors:** Dianbo Yao, Songlin Peng, Chaoliu Dai

**Affiliations:** 1 Department of Hepatobiliary and Splenic Surgery, Shengjing Hospital, China Medical University, Shenyang 110004, Liaoning Province, China

**Keywords:** Hepatocyte nuclear factor 4alpha, Mesenchymal epithelial transition, E-cadherin, Metastasis, Hepatocellular carcinoma

## Abstract

**Background:**

Mesenchymal–epithelial transition (MET) is now suggested to participate in the process of metastatic tumor formation. However, in hepatocellular carcinoma (HCC) the process is still not well revealed.

**Methods:**

Paraffin-embedded tissue samples were obtained from 13 patients with HCC in Shengjing Hospital of China Medical University. The expression of E-cadherin, Fibronectin, N-cadherin, Vimentin, Hepatocyte nuclear factor 4alpha (HNF4alpha), Snail and Slug was assessed in primary tumors and their corresponding metastases by immunohistochemical staining. Next, the expression of HNF4alpha and E-cadherin in four HCC cell lines was examined. Furthermore, SK-Hep-1 cells were transfected with human HNF4alpha expression vector, and the change of E-cadherin expression was assessed.

**Results:**

45.2% (14/31) of the lesions in the metastases showed increased E-cadherin expression compared with the primaries, suggesting the possible occurrence of MET in metastatic tumor formation of HCC, as re-expression of E-cadherin is proposed to be the important hallmark of MET. The occurrence of MET was also confirmed by the reduced expression of Fibronectin (54.8%, 17/31), N-cadherin (38.7%, 12/31) and Vimentin (61.3%, 19/31) in the metastases. 45.2% (14/31) of the lesions in the metastases also showed increased HNF4alpha expression, and 67.7% (21/31) and 48.4% (15/31) of metastases showed decreased Snail and Slug expression respectively. Statistical results showed that the expression of HNF4alpha was positively related with that of E-cadherin, and negatively correlated with that of Snail, Slug and Fibronectin, suggesting that the expression change of the MET markers in the metastatic lesions might be associated with HNF4alpha. Among the four HCC cell lines, both HNF4alpha and E-cadherin expressed high in Hep3B and Huh-7 cells, but low in SK-Hep-1 and Bel-7402 cells. Furthermore, the expression of E-cadherin increased accordingly when SK-Hep-1 cells were transfected with human HNF4alpha expression vector, further confirming the role of HNF4alpha in the regulation of E-cadherin expression.

**Conclusions:**

Our clinical observations and experimental data indicate that HNF4alpha might play a crucial role in the metastatic tumor formation of HCC, and the mechanism may be related with the process of phenotype transition.

## Background

Hepatocellular carcinoma (HCC) is one of the most common cancers worldwide, and the mortality rate is rather high [1]. Nowadays, surgical resection or liver transplantation remains the main and effective treatment throughout the world [2]. However, though great improvements have been made in the field of operative surgery, the long-term survival remains unsatisfied, mainly due to postoperative recurrence or metastasis [3]. Hence, investigation of the molecular mechanism of metastasis and recurrence would provide for the improvements of prognosis for the patients with HCC.

Recently, it has been demonstrated that mesenchymal–epithelial transition (MET) plays an important role in the metastasis of several kinds of tumors [4], while the experimental data supporting the role of MET in HCC are still limited. In 1996, Osada T proved that epithelial phenotype and E-cadherin played an important role in the process of intrahepatic metastasis of HCC, via two HCC cell sublines with different metastatic abilities, Li7HM and Li7NM [5]. However, further research was not carried out. In addition, Asayama Y performed an immunohistochemical study on 8 cases of HCC and its intrahepatic metastases, and found that the expression of E-cadherin and beta-catenin of intrahepatic metastases was similar to or even higher than those of primary lesions, suggesting that E-cadherin and beta-catenin might be significantly correlated with the metastatic tumor formation of HCC [6]. As the re-expression of E-cadherin is proposed to be the important hallmark of MET [7], it could be speculated that the MET might also play an important role in metastatic tumor formation of HCC.

The hepatocyte nuclear factor 4alpha (HNF4alpha) is a member of the steroid hormone receptor family, and plays an important role in regulation of hepatic gene expression [8]. Recently, it was reported that HNF4alpha is essential for morphological and functional differentiation of hepatocytes [9]. The genome-scale chromatin immunoprecipitation assay showed that HNF4alpha could bind to the promoters of nearly half of the genes that are expressed in the mouse liver, including cell adhesion and junctional proteins that allow the hepatic cells to form a polarized epithelium [10], suggesting that HNF4alpha should be a dominant regulator of the epithelial phenotype. In addition, it was demonstrated that the expression of HNF4alpha in dedifferentiated rat hepatoma H5 cells could result in re-expression of cytokeratin proteins and partial reestablishment of E-cadherin production [11]. Forced re-expression of HNF4alpha in a dedifferentiated hepatoma cell line was also shown to induce the cells to re-form junctions and express hepatocyte marker genes [12]. So, it could be speculated that HNF4alpha may be also a main regulator of E-cadherin expression in HCC, or even an important participant in the metastatic tumor formation of HCC. Besides, recently HNF4alpha was found to be able to directly inhibit transcription of the EMT master regulatory genes Snail and Slug and of several mesenchymal markers, and it might be just by this mechanism that HNF4alpha could induce the MET [13].

Therefore, in this study we aimed to experimentally examine whether HNF4alpha take part in the metastatic tumor formation of HCC and its relationship with the MET markers. An immunohistochemical study of the expression of E-cadherin and some other markers of MET (including Vimentin, Fibronectin and N-cadherin) revealed the increased E-cadherin, and reduced Fibronectin, N-cadherin and Vimentin expression in the metastases compared with the primaries, suggesting that the MET occurred. The expression of HNF4alpha was similarly increased with that of E-cadherin in the metastases, and the expression of Snail and Slug in the metastases was significantly reduced compared with the primaries. In addition, it was showed that the expression of HNF4alpha was positively related with that of E-cadherin, but negatively related with the expression of Fibronectin, Snail and Slug in primary tumors and metastatic lesions of HCC. These suggested that HNF4alpha might also play a crucial role in the metastatic tumor formation of HCC, and might possibly be related with the expression change of E-cadherin, Fibronectin, Snail and Slug. Furthermore, the examination of HNF4alpha and E-cadherin expression in four HCC cell lines revealed again the association of E-cadherin expression with the HNF4alpha expression, and it was found that increased expression of HNF4alpha in SK-Hep-1 cells could result in an increased expression of E-cadherin, confirming the role of HNF4alpha in the regulation of E-cadherin expression. What we found suggested that HNF4alpha might play an important role in the metastatic tumor formation of HCC, and it might be related with the expression change of MET markers, or even the MET in the metastases.

## Methods

### Cell culture

American Type Culture Collection (ATCC) cell lines, Hep3B and SK-Hep-1 cells were cultured in MEM medium with 10% fetal bovine serum. Bel-7402 cells were cultured in RPMI-1640 medium with 10% fetal bovine serum, while Huh-7 cells were cultured in DMEM medium with 10% fetal bovine serum. All cells were incubated at 37°C in 5% CO2.

### Immunohistochemistry

Paraffin-embedded patient samples were obtained from Shengjing Hospital of China Medical University. Informed consent was obtained directly from individual patients and subject’s relatives, and the experimental protocols were reviewed and approved by the Ethics Committee of the hospital (reference number: 2012PS34K). The staining procedures were performed according to the manufacturer’s protocols. Immunostaining was performed on 4 μm paraffin-embedded tissue sections. The slides were deparaffinized in xylene and dehydrated in a graded ethanol series, and the sections underwent antigen retrieval in citrate solution. Endogenous peroxidase was blocked with 3% hydrogen peroxide, and the sections were washed with phosphate-buffered saline. After blocking, they were incubated overnight with E-cadherin (1:200, sc-8426, Santa Cruz Biotechnology, Santa Cruz, CA), Vimentin (1:100, Santa Cruz Biotechnology, Santa Cruz, CA), Fibronectin(1:100, sc-18825, Santa Cruz Biotechnology, Santa Cruz, CA), N-cadherin (1:100, Santa Cruz Biotechnology, Santa Cruz, CA), HNF4alpha (1:150, BS2983, Bioworld Technology), Snail (1:50, ab135708, Abcam Technology) or Slug (1:100, #9585, Cell Signaling Technology) primary antibodies. Antigen staining was performed using DAB horseradish peroxidase color development kit and then counterstained with hematoxylin. The immunoreactivity of proteins in each tissue core was assessed independently by two experienced pathologists for staining intensity (0 absent, 1 weak, 2 intermediate, 3 strong staining).

### Western blot

Cell lysate proteins were resolved on 8% sodium dodecyl sulfate polyacrylamide gel electropheresis (SDS-PAGE) and transferred to PVDF membranes. After blocking, membranes were incubated with primary antibodies against E-cadherin (1:250, sc-8426, Santa Cruz Biotechnology, Santa Cruz, CA), HNF4alpha (1:250, sc-8987, Santa Cruz Biotechnology, Santa Cruz, CA) and β-actin (1:200, sc-69879, Santa Cruz Biotechnology, Santa Cruz, CA), followed by incubation with peroxidase-conjugated secondary antibodies and chemiluminescence detection.

### Immunofluorescence analysis

2×10^4^ Hep3B or SK-Hep-1 cells on cover glass were washed and fixed with 4% paraformaldehyde at 4°C for 2 h. The cells were treated with 0.1% Triton X-100 for 15 min at room temperature and were blocked for 30 min with blocking buffer (10% BSA in PBS) at room temperature. The cells were incubated for overnight with E-cadherin (1:50, sc-7870, Santa Cruz Biotechnology, Santa Cruz, CA), HNF4alpha (1:150, sc-8987, Santa Cruz Biotechnology, Santa Cruz, CA) and vimentin (1:100, BS1855, Bioworld Technology) primary antibodies, followed by incubation with the appropriate fluorophore-labeled secondary antibody for 1 h, and then for 15 min with 10 μM Heochst 33342 at room temperature. Visualization was performed on an Olympus fluorescence microscope.

### Transient transfection

Cells were seeded as 6×10^5^ cells per well of 6-well plate. After about 16 h, cells were transfected with 4 μg of pTarget-HNF4alpha plasmids (a kind gift from Kobayashi K [14]), and 10 μl of Lipofectamine2000 (Invitrogen, CA) according to the manufacturer’s protocol. After 48 h transfection, the cells were collected for detection.

### Stable transfection

Cells were seeded as 2×10^5^ cells per well of 24-well plate. After 16 h, cells were transfected with 0.8 μg of pTarget-HNF4alpha plasmids and 2 μl of Lipofectamine2000. After 24 h, the cells were cultured in 900 μg/ml G418 to select for stable transfection. After about 2 weeks, the stable clones were picked for growth on plates, and maintained in 450 μg/ml G418 for the detection of ideal clones.

### Statistical analysis

The Wilcoxon matched pairs test was used to test the differences of E-cadherin, Fibronectin, N-cadherin, Vimentin, HNF4alpha, Snail or Slug expression between primary liver tumors and their corresponding metastases. Spearman correlation analysis was used to test the correlation of HNF4alpha with E-cadherin, Vimentin, Fibronectin, N-cadherin, Snail and Slug expression in primary liver tumors and their corresponding metastases. All p values reported are two-sided, and the significance level was set at less than 0.05. The analyses were performed with the SPSS 13.0 statistical software program.

## Results

### The expression change of the MET markers in primary HCC lesions and their corresponding metastases

A few studies have examined E-cadherin expression in the primary tumor and distant metastases, including breast or prostate cancer specimens, and the role of E-cadherin in metastatic tumor formation has been gradually revealed [15,16]. To conduct our survey focusing on metastases of HCC, we obtained specimens of primary tumors and the corresponding metastases from 13 patients with HCC. The metastatic sites from which the lesions could be obtained included the lymph nodes (24 lesions), stomach (4 lesions), and peritoneum (3 lesions). Both primary tumor and metastases were immunostained for E-cadherin. E-cadherin positive cells were counted based on high intensity membrane or cytoplasmic staining, as E-cadherin expression was not always localized to the membrane [16].

**Figure 1:**
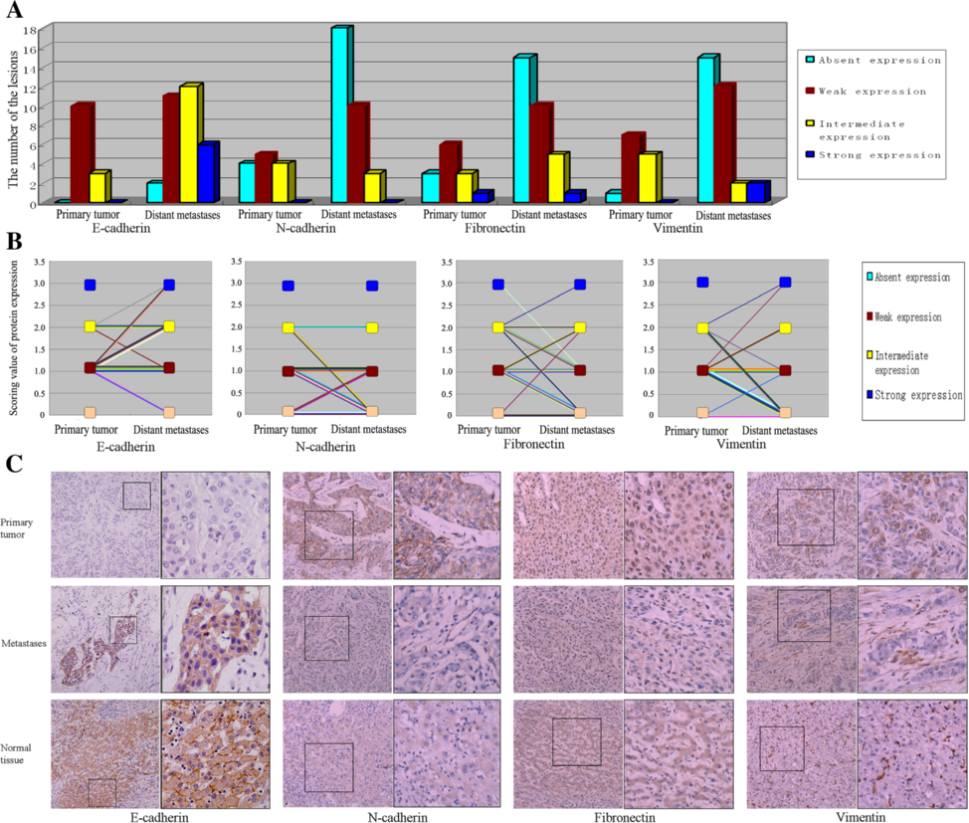
**The expression of MET marks in primary tumors and their corresponding metastases suggested the occurrence of MET in the metastases. A)** The number of the lesions with different staining intensity of the MET marks (including E-cadherin, N-cadherin, Fibronectin and Vimentin) in the primary tumors and their corresponding metastases. **B)** The expression change of MET marks in the metastases compared with their corresponding primary tumors (increased E-cadherin, decreased N-cadherin, Fibronectin and Vimentin expression), marking via the lines, showed the occurrence of MET in the metastases. **C)** The examples of the cases with increased E-cadherin, decreased N-cadherin, Fibronectin and Vimentin expression in the metastases compared with their primary tumors were showed. In the normal tumor-adjacent tissue, the expression of E-cadherin was mainly strong, but the expression of N-cadherin, Fibronectin and Vimentin was usually negative.

Overall, the expression of E-cadherin in the primary tumors showed weak in 10 cases, intermediate in the other 3 cases, while the expression of E-cadherin in the metastases showed absent in 2 lesions, weak in 11 lesions, intermediate in 12 lesions and strong in 6 lesions (Figure 1A). It was found that 45.2% (14/31) of the lesions in the metastases showed increased E-cadherin expression compared with the primaries, while only 12.9% (4/31) showed decreased E-cadherin expression (Figure 1B). The expression of E-cadherin in metastases was significantly increased (p<0.05 by Wilcoxon paired analyses), suggesting that the E-cadherin might play an important role in the formation of HCC metastases.

A closer examination of the specimens revealed striking differences of E-cadherin expression between the primary tumor and the metastases in some cases, with the primary tumor wholly negative and the metastases mostly positive for E-cadherin expression (Figure 1C). Interestingly, though the metastases mostly showed only increased cytoplasmic expression of E-cadherin, in some cases, the metastases also showed recovered membranous expression of E-cadherin (Figure 1C).

E-cadherin is now recognized as an indicator of mesenchymal to epithelial reverting transitions during the metastatic seeding of disseminated carcinomas [7]. In order to further investigate the phenotype transitions during the metastasis of HCC, we analyzed the expression pattern of some more markers of MET, including Vimentin, Fibronectin and N-cadherin. Immunostaining analysis revealed that 38.7% (12/31) of metastases showed decreased N-cadherin expression, and 54.8% (17/31) of metastases showed decreased Fibronectin expression (Figure 1B), 61.3% (19/31) of metastases showed decreased Vimentin expression (Figure 1B). Now, the significantly decreased expression of N-cadherin, Fibronectin and Vimentin (p<0.05 by Wilcoxon paired analyses) comfirmed the occurrence of MET in the metastases.

In addition, among the 13 cases of primary tumors, normal tumor-adjacent tissues could be found in 11 cases. It was found that comparing with these normal tumor-adjacent tissues, the expression of E-cadherin in the primary tumors was all down-regulated (Figure 1C), and in the metastases most of E-cadherin expression were also slightly reduced, though in some cases (19.4%, 6/31) the expression of E-cadherin in the metastases was similar with that in the normal tissues (Figure 1C). For the other markers, compared with these normal tumor-adjacent tissues, most of the Fibronectin (76.9%, 10/13), N-cadherin (69.2%, 9/13), and Vimentin (92.3%, 12/13) expression in the primaries appeared increased, while most of the metastases (48.4%, 15/31, 58.1%, 18/31, 48.4%, 15/31 respectively for Fibronectin, N-cadherin and Vimentin) showed a low-expressed appearance, similarly with the normal tissues (Figure 1C).

### The expression of HNF4alpha increased in HCC metastases and was closely related with the expression of MET markers, Snail, and Slug

**Figure 2:**
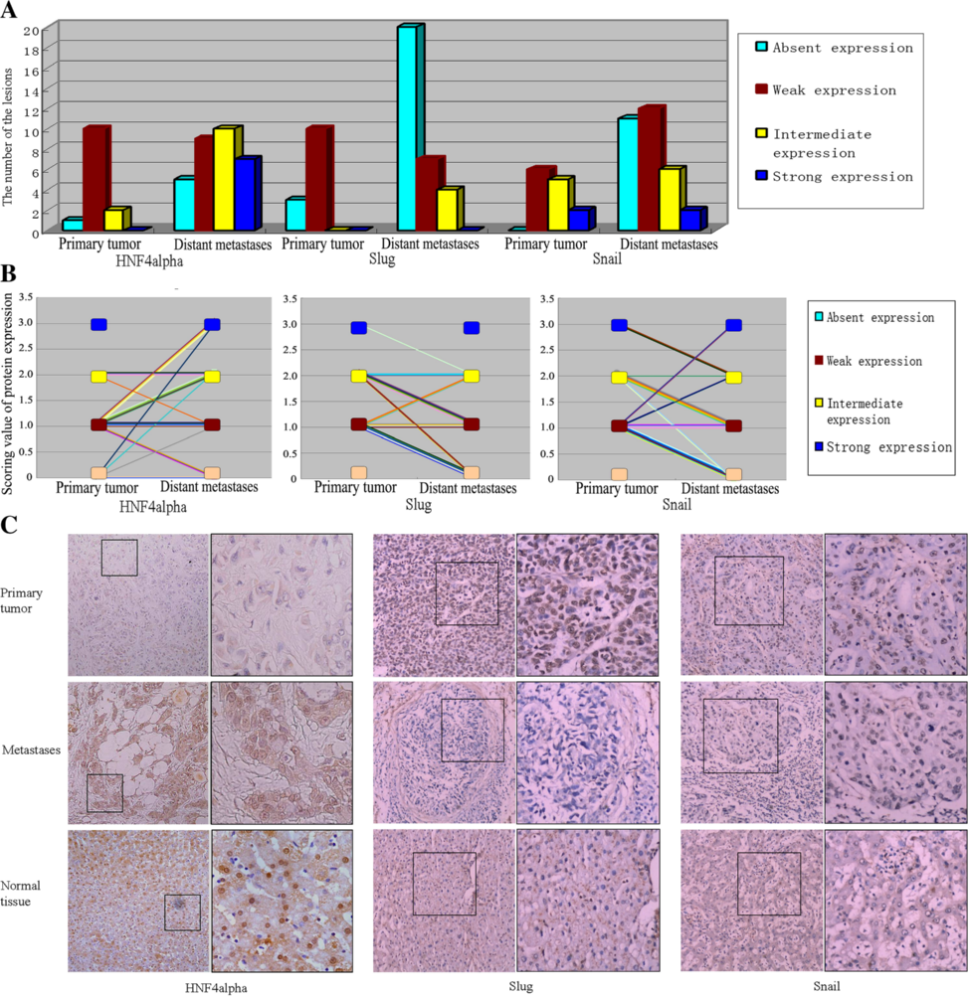
**The expression of HNF4alpha, slug and snail in primary tumors and their corresponding metastases. A)** The number of the lesions with different staining intensity of HNF4alpha, Slug and Snail in the primary tumors and its metastases. **B)** The expression change of HNF4alpha, Slug and Snail in the metastases compared with their corresponding primary tumors, marking via the lines, showed the role of HNF4alpha, Slug and Snail in the metastases. **C)** The examples of the cases with increased HNF4alpha, decreased Slug and Snail expression in the metastases compared with their primary tumors were showed. In the normal tumor-adjacent tissue, the expression of HNF4alpha was mainly strong, while the expression of Slug and Snail was usually negative.

To reveal the regulatory mechanism of E-cadherin expression, both the primary tumors and metastases were also immunostained for HNF4alpha, which might be a main regulator of E-cadherin expression in HCC. HNF4alpha immunoreactivity was similarly determined. Each tissue core was assessed for nuclear or cytoplasmic staining intensity (absent, weak, intermediate or strong), as the expression position of HNF4alpha in tumor would also be changed [17]. Overall, the expression of HNF4alpha in the primary tumors showed absent in 1 case, weak in 10 cases and intermediate in the other 2 cases, while expression of HNF4alpha in the metastases showed absent in 5 lesions, weak in 9 lesions, intermediate in 10 lesions and strong in the other 7 lesions (Figure 2A). It was found that 45.2% (14/31) of the lesions showed increased HNF4alpha expression in the metastases compared with the primaries, while only 12.9% (4/31) showed decreased HNF4alpha expression in the metastases (Figure 2B). The difference of HNF4alpha expression in primary tumors and their corresponding metastatic lesions was also significant (p<0.05 by Wilcoxon paired analyses).

Similarly, it could also be found that in some cases with the primary tumor negative expression of HNF4alpha, the metastases showed obvious HNF4alpha expression (Figure 2C). In addition, comparing with the normal tissues, the expression of HNF4alpha in either the primary tumors or the metastases was mostly down-regulated (Figure 2C), with only a small part of metastases (22.6%, 7/31) showing a similar expression with the normal tissues.

In our study, the expression of two EMT master regulatory genes, Snail and Slug, whose transcription was introduced to be able to be directly inhibited by HNF4alpha, was also detected in the primary HCC tumor and corresponding distant metastases. 67.7% (21/31) and 48.4% (15/31) of cases showed decreased Snail and Slug expression in the metastases compared with the primaries, while only 12.9% (4/31) and 16.1% (5/31) showed increased Snail and Slug expression in the metastases (Figure 2B). The difference of Snail and Slug expression in primary tumors and their corresponding metastatic lesions was also significant (p<0.05 by Wilcoxon paired analyses). In addition, the expression of Snail and Slug in normal tumor-adjacent tissues was mostly weak or negative (Figure 2C).

The expression of HNF4alpha, Snail, Slug, E-cadherin, Fibronectin, N-cadherin and Vimentin was all detected in the primary tumors and their corresponding metastases. It was found that comparing with the normal tissues, the expression of both HNF4alpha and E-cadherin in primary tumors was mostly decreased, while the expression of Snail, Slug, Fibronectin, N-cadherin and Vimentin was mostly increased. Besides, both E-cadherin and HNF4alpha mostly showed increased expression in the metastases compared with the primary tumors, while Snail, Slug, Fibronectin, N-cadherin and Vimentin mostly showed decreased expression in the metastases. Spearman correlation analysis showed that the expression of Snail and Slug in the primary tumors and their corresponding metastases was negatively correlated with the expression of E-cadherin, and positively correlated with that of Fibronectin, N-cadherin and Vimentin (Additional file 1: Table S1), confirming again that Snail and Slug were the EMT master regulatory genes. In addition, as previous studies showed that HNF4alpha might induce the MET through inhibiting the transcription of EMT master regulatory genes Snail and Slug, it could be speculated that the expression of Snail, Slug, E-cadherin, Fibronectin, N-cadherin and Vimentin might be closely associated with that of HNF4alpha. Spearman correlation analysis showed that the expression of HNF4alpha in the primary tumors and their corresponding metastases was significantly positively correlated with the expression of E-cadherin, and negatively correlated with the expression of Snail, Slug and Fibronectin (Additional file 1: Table S1), primarily demonstrating the conjecture.

### The important role of HNF4alpha in the regulation of E-cadherin expression in HCC cells

**Figure 3:**
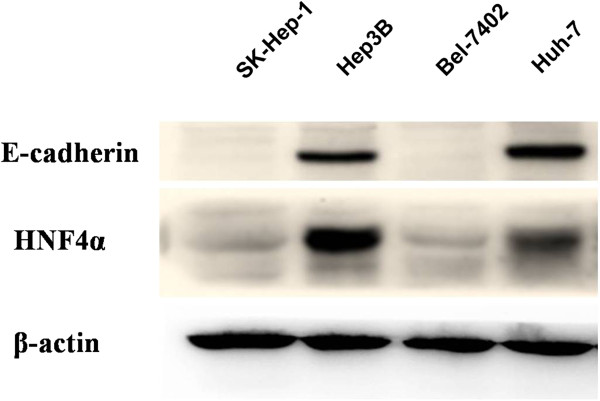
**Correlation of the E-cadherin and HNF4alpha expression showed in four HCC cell lines.** The expression of E-cadherin and HNF4alpha was detected in four HCC cell lines by western blot examination. It was showed that E-cadherin and HNF4alpha protein expression were both strong in Hep3B and Huh-7 cells, but weak in SK-hep-1 and BEL-7402 cells, showing the correlation of the E-cadherin and HNF4alpha expression.

**Figure 4:**
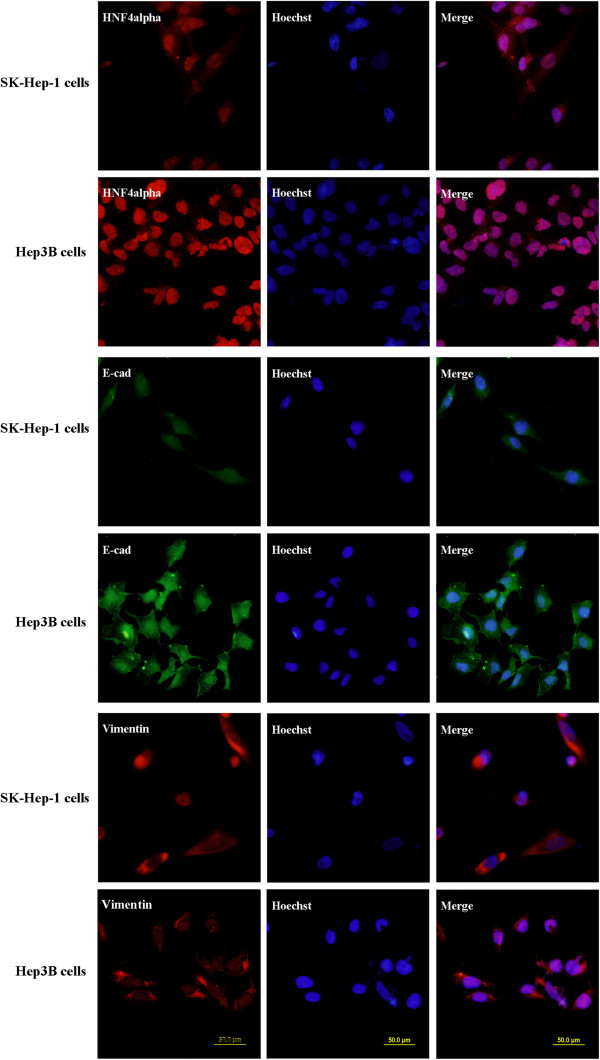
**The expression of HNF4alpha, E-cadherin and vimentin in SK-hep-1 and Hep3B cells.** Immunofluorescence examination showed again the expression of E-cadherin and HNF4alpha in Hep3B cells was stronger than that in SK-hep-1 cells. In addition, it was found that the expression of vimentin in SK-hep-1 cells is similar with that in Hep3B cells.

The results of immunohistochemistry detection on HCC specimens showed that the expression of E-cadherin was closely correlated with that of HNF4alpha. Next, four HCC cell lines were also detected for the expression of E-cadherin and HNF4alpha via western blot examination. It was showed that E-cadherin and HNF4alpha protein expression were both strong in Hep3B and Huh-7 cells, but weak in SK-hep-1 and BEL-7402 cells (Figure 3). These showed again the possibly close correlation of the E-cadherin and HNF4alpha expression. Besides, immunofluorescence examination in the SK-hep-1 and Hep3B cells confirmed the result of western blot examination (Figure 4). Furthermore, the expression of vimentin was also detected, and it was found that the expression difference of the vimentin in the two HCC cell lines was not obvious (Figure 4).

**Figure 5:**
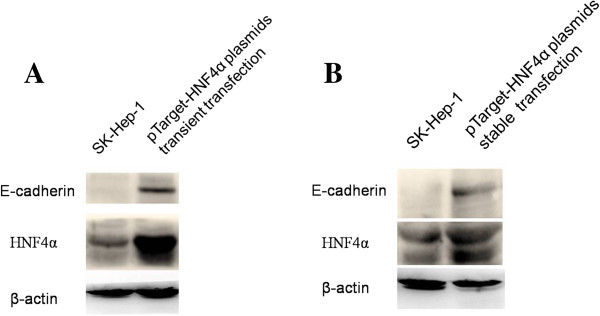
**HNF4alpha regulated the expression of E-cadherin in HCC cells.** The SK-Hep-1 cells were transfected with HNF4alpha-expression plasmid. As the expression of HNF4alpha increased, the expression of E-cadherin correspondingly increased. **A)** Transient transfection. **B)** Stable transfection.

Next, the SK-Hep-1 cells in which E-cadherin expression was undetected via western blot detection were selected for the transfection, to reveal the role of HNF4alpha in the regulation of E-cadherin expression. The SK-Hep-1 cells were transfected with HNF4alpha-expression plasmid. The expression of HNF4alpha was confirmed to increase, and the expression of E-cadherin was revealed to increase correspondingly (Figure 5), confirming that HNF4alpha play an important role in the regulation of E-cadherin expression, in the human liver cancer cells.

## Discussion

Though much more progress has been got during the researches for the metastasis of tumors, the mechanism of tumor metastases formation, which is the later stage of metastasis, is rarely revealed. One important reason for this is that the clinical metastases specimens are much more difficult to get. Nowadays, some researches that were carried out for tumor metastases revealed that many metastatic lesions and their corresponding primary tumors shared a similar epithelial nature. Some metastases of a number of carcinomas, including prostatic cancer, breast carcinoma, colorectal cancer, ovarian cancer and pulmonary cancer, were even less dedifferentiated than their corresponding primary tumors [4]. As for HCC, it was revealed in the Asayama Y’s study that the expression of E-cadherin in intrahepatic metastases was similar with those of primary lesions in most cases, while in two cases with reduced E-cadherin expression in the primary sites, the expression of E-cadherin in intrahepatic metastases was even preserved [6]. Based on the observations of increased E-cadherin expression in metastases compared with primary tumors, they proposed that preserved or recovered E-cadherin expression might be of beneficial effect, possibly for re-establishing the tissue architecture at the metastatic site [6].

In our study, we surveyed a set of matched primary and metastatic tumors, for the expression of E-cadherin in the HCC specimens. It was found that 45.2% of metastases expressed increased E-cadherin compared with the primary tumors, which mostly exhibited low or negative E-cadherin expression, suggesting again the possible role of E-cadherin in the metastases formation of HCC. In addition, as the re-expression of E-cadherin is proposed to be the important hallmark of MET [7], it also suggested that the MET might play an important role in metastatic tumor formation of HCC. In our study, some other marks of MET, including vimentin, fibronectin, N-cadherin, were also examined. It was found that 38.7%, 54.8% and 61.3% of metastases showed decreased expression of N-cadherin, Fibronectin and Vimentin respectively. These results further suggested the possible occurrence of MET in the metastases of HCC.

EMT represents a fundamentally important process that is conducive to tumor dissemination [18,19]. EMT in cancer progression and metastasis has been widely studied through in vitro cell culture and in vivo animal models of cancer progression [20]. The downregulation or silencing of E-cadherin, which is the key molecule for the maintenance of epithelial integrity and polarized function, is the critical event of EMT. It is thought in the EMT theory that epithelial cells could acquire fibroblast-like properties and exhibit reduced cell-cell adhesion and increased motility via EMT [21]. Whereas, at present, the results of tumor metastases examination showed that the metastases did not only compose of mesenchymal tumor cells, and this seemed to be in contradiction with the theory of EMT. Now, it is proposed that the process of MET would occur in later stages of tumor metastasis, possibly to fit into the metastatic process, explaining why the distant metastases had similar phenotype with the primary lesions [22-24].

As for the possible mechanism that the MET contributes to the metastatic tumor formation, it was thought that the re-expressed E-cadherin might contribute to the formation of metastatic tumors by helping the establishment of organizational structure in metastases [25]. What is more, the E-cadherin was also found to be able to activate intracellular proliferation and survival signals by activating the survival-associated mitogen activated protein kinase (MAPK) and Akt/PKB cascades in the distance microenvironment [26]. Certainly, the exact related mechanism of the MET in the metastases is still waiting for further revelation [4].

In this study, we also detected the expression of HNF4alpha, which was found to possibly play an important role in the expression regulation of E-cadherin in HCC [10,11], in the specimens of HCC and its metastases. It was revealed that 45.2% of HCC metastatic lesions also expressed higher HNF4alpha than their corresponding primary foci, and the positive expression of HNF4alpha and E-cadherin in HCC and its metastases was obviously relevant, primarily suggesting that HNF4alpha might also play a crucial role in the metastatic tumor formation of HCC, and might be related with the expression change of E-cadherin in primary hepatic tumors or its metastases.

HNF4alpha has been previously recognized as a main regulator of E-cadherin expression. It was found that HNF4alpha could induce increased expression of E-cadherin in two HCC cell lines with epithelial phenotypes, Hep3B and HepG2, proving HNF4alpha could regulate the expression of E-cadherin in human hepatoma cells [27]. Now, the role of HNF4alpha in the expression regulation of E-cadherin in the HCC cells with mesenchymal phenotypes was further confirmed in our study via transfection, as it was found that the expression of E-cadherin increased accordingly when SK-Hep-1 cells were transfected with human HNF4alpha expression vector. Though in our study, it was found that the transfected cells were not epithelialized (data not showed), the role of HNF4alpha on the induction of MET could not be easily denied. In the Späth GF’s study, it was also found that HNF4 could only result in reexpression of cytokeratin proteins and partial reestablishment of E-cadherin production, and the cells were not epithelialized. But they found that only the transfectants are competent to respond to the synthetic glucocorticoid dexamethasone, which induces the second step of morphogenesis, including formation of the junctional complex and expression of a polarized cell phenotype, suggesting that HNF4 should be an important part of the MET inducers. In addition, it was also introduced that the expression of HNF4alpha could directly induce the MET in fibroblasts (NIH3T3 cells) [9], and Utilizing rat hepatoma cell lines, Natalia L Lazarevich et al also proved that dedifferentiated hepatoma cells can be induced to transform to the epithelial phenotype by re-expression of HNF4alpha [28]. These findings suggest that HNF4alpha should be an important inducer of the MET in HCC cells, or in some cases, it acts as an important part of the MET inducers, possibly by regulating the expression of E-cadherin. Now, combining with what we have found in the clinical specimens, it could be speculated that in HCC, MET might also play an important role in metastatic tumor formation, and HNF4alpha might just be the important inducer of the MET, or act as an important part of the MET inducers. Certainly, the exact role of HNF4alpha in the metastatic tumor formation of HCC is still needed for further confirmed, maybe through the model of experimental tumor metastasis in vivo.

Now, the role of HNF4alpha in the expression regulation of E-cadherin has been further confirmed in our study. As for the mechanism, recently, it was found that HNF4alpha could directly inhibit the transcription of EMT master regulatory genes Snail, Slug and several mesenchymal markers [13]. We think that it might possibly be the answer, as zinc finger transcription factors of the Snail family have been characterized as key repressors of the E-cadherin gene, by acting through interaction with specific E-boxes in the proximal promoter [29]. In our study, it was found that the expression of HNF4alpha in the primary tumors and their corresponding metastases was significantly positively correlated with the expression of E-cadherin, and negatively correlated with the expression of Snail and Slug, primarily demonstrated our conjecture. In addition, it was also found in previous study that Snail could repress the transcription of the HNF4alpha gene through a direct binding to its promoter [30]. So, the regulation relationship between the HNF4alpha and Snail is still needed for further research.

In our study, it was also found that not all metastases were detected to express increased E-cadherin, or decreased expression of N-cadherin, Fibronectin and Vimentin. As for this, we consider that it might be that a second EMT occurred within the metastatic lesions, for further invasion and disseminations. In the study of Chao Y, it was found that E-cadherin expression in prostate cancer metastases was inversely correlated with size of metastasis, with increased E-cadherin expression in small metastases compared with large ones [15], suggesting that the EMT was likely occurring in the metastases.

In addition, we also found that in normal tumor-adjacent tissues both HNF4alpha and E cadherin was high expressed, while Snail, Slug, Fibronectin, N-cadherin and Vimentin were mostly low expressed. Compared with the normal tumor-adjacent tissues, the expression of HNF4alpha and E cadherin in either primary HCC or their metastases was mainly reduced, and the expression of Snail, Slug, Fibronectin and N-cadherin was mainly increased, suggesting that the reduced expression of HNF4alpha and E cadherin, and increased expression of Snail, Slug, Fibronectin and N-cadherin may be associated with the occurrence or progression of HCC. This confirmed previous researches [28,31,32]. Recently, the findings in rat liver cancer model studies suggested that the transcription inhibition of HNF4alpha was related with tumor progression and dedifferentiation, and exogenous expression of HNF4alpha could make the chemical-induced rat dedifferentiated carcinoma reverse its malignant phenotype [33], proving that reduction of HNF4alpha expression played an important role in HCC progression, and was closely related with EMT. Therefore, combining with above results, we could suppose that the alterations of HNF4alpha expression may play important roles in occurrence of liver tumors, tumor progression and metastatic tumor formation. The expression of HNF4alpha slightly reduced during the occurrence of HCC, further decreased in HCC progress, while partly restored during tumor metastases formation.

In the Paget’s seed and soil hypothesis, it was postulated that cancer cells, or the “seeds”, will only grow in a specific microenvironment, or “soil” [34]. The reversible phenotypic of cancer cells during EMT and MET may therefore be one way by which cancer cells can adapt to the foreign soil of ectopic organ microenvironments. To reveal the mechanisms of the EMT and MET process may help us interfere this dynamic process, or even further inhibit the metastasis. Now, our clinical and experimental data indicate that HNF4alpha might play a crucial role in this dynamic process during the metastasis of HCC, and the related mechanism is worth further studying.

## Conclusions

In summary, our data indicated that HNF4alpha might play a crucial role in the process of phenotype transition during the metastasis of HCC and it may be related with the process of phenotype transition. Apparently, our study preliminarily demonstrated the important role of HNF4alpha, and might provide for further study of related mechanisms in the metastatic tumor formation of HCC.

## Abbreviations

MET: Mesenchymal–epithelial transition; HCC: Hepatocellular carcinoma; HNF4alpha: Hepatocyte nuclear factor 4alpha.

## Competing interests

The authors declared that they have no competing interests.

## Authors’ contributions

DY designed the study, performed the experiments and drafted the manuscript; SP performed the experiments; CD supervised the experiments and edited the manuscript. All authors read and approved the final manuscript.

## Pre-publication history

The pre-publication history for this paper can be accessed here:

http://www.biomedcentral.com/1471-2407/13/432/prepub

## Supplementary Material

Additional file 1: Table S1 Correlation between HNF4alpha, Snail, Slug, E-cadherin, Fibronectin, N-cadherin and Vimentin in primary tumors and their corresponding metastases samples. Click here for file

## References

[B1] Herszényi L Tulassay Z Epidemiology of gastrointestinal and liver tumors Eur Rev Med Pharmacol Sci 2010 14 249 258 20496531

[B2] Forner A Llovet JM Bruix J Hepatocellular carcinoma Lancet 2012 379 1245 1255 10.1016/S0140-6736(11)61347-0 22353262 PMC13537732

[B3] Tung-Ping Poon R Fan ST Wong J Risk factors, prevention, and management of postoperative recurrence after resection of hepatocellular carcinoma Ann Surg 2000 232 10 24 10.1097/00000658-200007000-00003 10862190 PMC1421103

[B4] Yao D Dai C Peng S Mechanism of the mesenchymal-epithelial transition and its relationship with metastatic tumor formation Mol Cancer Res 2011 9 1608 1620 10.1158/1541-7786.MCR-10-0568 21840933

[B5] Osada T Sakamoto M Ino Y Iwamatsu A Matsuno Y Muto T Hirohashi S E-cadherin is involved in the intrahepatic metastasis of hepatocellular carcinoma Hepatology 1996 24 1460 1467 10.1002/hep.510240627 8938181

[B6] Asayama Y Taguchi Ki K Aishima Si S Nishi H Masuda K Tsuneyoshi M The mode of tumour progression in combined hepatocellular carcinoma and cholangiocarcinoma: an immunohistochemical analysis of E-cadherin, alpha-catenin and beta-catenin Liver 2002 22 43 50 11906618 10.1046/j.0106-9543.2001.01580.x

[B7] Wells A Yates C Shepard CR E-cadherin as an indicator of mesenchymal to epithelial reverting transitions during the metastatic seeding of disseminated carcinomas Clin Exp Metastasis 2008 25 621 628 10.1007/s10585-008-9167-1 18600305 PMC2929356

[B8] Odom DT Zizlsperger N Gordon DB Bell GW Rinaldi NJ Murray HL Volkert TL Schreiber J Rolfe PA Gifford DK Fraenkel E Bell GI Young RA Control of pancreas and liver gene expression by HNF transcription factors Science 2004 303 1378 1381 10.1126/science.1089769 14988562 PMC3012624

[B9] Parviz F Matullo C Garrison WD Savatski L Adamson JW Ning G Kaestner KH Rossi JM Zaret KS Duncan SA Hepatocyte nuclear factor 4alpha controls the development of a hepatic epithelium and liver morphogenesis Nat Genet 2003 34 292 296 10.1038/ng1175 12808453

[B10] Battle MA Konopka G Parviz F Gaggl AL Yang C Sladek FM Duncan SA Hepatocyte nuclear factor 4alpha orchestrates expression of cell adhesion proteins during the epithelial transformation of the developing liver Proc Natl Acad Sci USA 2006 103 8419 8424 10.1073/pnas.0600246103 16714383 PMC1482507

[B11] Späth GF Weiss MC Hepatocyte nuclear factor 4 provokes expression of epithelial marker genes, acting as a morphogen in dedifferentiated hepatoma cells J Cell Biol 1998 140 935 946 10.1083/jcb.140.4.935 9472044 PMC2141753

[B12] Lazarevich NL Al’pern DV Hepatocyte nuclear factor 4 (HNF4) in epithelial development and carcinogenesis Mol Biol (Mosk) 2008 42 786 797 18988528

[B13] Santangelo L Marchetti A Cicchini C Conigliaro A Conti B Mancone C Bonzo JA Gonzalez FJ Alonzi T Amicone L Tripodi M The stable repression of mesenchymal program is required for hepatocyte identity: a novel role for hepatocyte nuclear factor 4alpha Hepatology 2011 53 2063 2074 10.1002/hep.24280 21384409 PMC6624426

[B14] Iwazaki N Kobayashi K Morimoto K Hirano M Kawashima S Furihata T Chiba K Involvement of hepatocyte nuclear factor 4 alpha in transcriptional regulation of the human pregnane X receptor gene in the human liver Drug Metab Pharmacokinet 2008 23 59 66 10.2133/dmpk.23.59 18305375

[B15] Chao Y Wu Q Acquafondata M Dhir R Wells A Partial mesenchymal to epithelial reverting transition in breast and prostate cancer metastases Cancer Microenviron 2012 5 19 28 10.1007/s12307-011-0085-4 21892699 PMC3343195

[B16] Chao YL Shepard CR Wells A Breast carcinoma cells re-express E-cadherin during mesenchymal to epithelial reverting transition Mol Cancer 2010 9 179 10.1186/1476-4598-9-179 20609236 PMC2907333

[B17] Chellappa K Jankova L Schnabl JM Pan S Brelivet Y Fung CL Chan C Dent OF Clarke SJ Robertson GR Sladek FM Src tyrosine kinase phosphorylation of nuclear receptor HNF4α correlates with isoform-specific loss of HNF4α in human colon cancer Proc Natl Acad Sci USA 2012 109 2302 2307 10.1073/pnas.1106799109 22308320 PMC3289305

[B18] Kalluri R Weinberg RA The basics of epithelial-mesenchymal transition J Clin Invest 2009 119 1420 1428 10.1172/JCI39104 19487818 PMC2689101

[B19] Thiery JP Acloque H Huang RY Nieto MA Epithelial-mesenchymal transitions in development and disease Cell 2009 139 871 890 10.1016/j.cell.2009.11.007 19945376

[B20] Wells A Chao YL Grahovac J Wu Q Lauffenburger DA Epithelial and mesenchymal phenotypic switchings modulate cell motility in metastasis Front Biosci 2011 16 815 837 10.2741/3722 PMC4003907 21196205

[B21] Thiery JP Sleeman JP Complex networks orchestrate epithelial mesenchymal transitions Nat Rev Mol Cell Biol 2006 7 131 142 10.1038/nrm1835 16493418

[B22] Chaffer CL Thompson EW Williams ED Mesenchymal to epithelial transition in development and disease Cells Tissues Organs 2007 185 7 19 10.1159/000101298 17587803

[B23] Hugo H Ackland ML Blick T Lawrence MG Clements JA Williams ED Thompson EW Epithelial—mesenchymal and mesenchymal—epithelial transitions in carcinoma progression J Cell Physiol 2007 213 374 383 10.1002/jcp.21223 17680632

[B24] Brabletz T To differentiate or not - routes towards metastasis Nat Rev Cancer 2012 12 425 436 10.1038/nrc3265 22576165

[B25] Yates CC Shepard CR Stolz DB Wells A Co-culturing human prostate carcinoma cells with hepatocytes leads to increased expression of E-cadherin Br J Cancer 2007 96 1246 1252 10.1038/sj.bjc.6603700 17406365 PMC2360137

[B26] Reddy P Liu L Ren C Lindgren P Boman K Shen Y Lundin E Ottander U Rytinki M Liu K Formation of E-cadherin-mediated cell-cell adhesion activates AKT and mitogen activated protein kinase via phosphatidylinositol 3 kinase and ligand-independent activation of epidermal growth factor receptor in ovarian cancer cells Mol Endocrinol 2005 19 2564 2578 10.1210/me.2004-0342 15928314

[B27] Yin C Lin Y Zhang X Chen YX Zeng X Yue HY Hou JL Deng X Zhang JP Han ZG Xie WF Differentiation therapy of hepatocellular carcinoma in mice with recombinant adenovirus carrying hepatocyte nuclear factor-4alpha gene Hepatology 2008 48 1528 1539 10.1002/hep.22510 18925631

[B28] Lazarevich NL Fleishman DI Tissue-specific transcription factors in progression of epithelial tumors Biochemistry (Mosc) 2008 73 573 591 10.1134/S0006297908050106 18605982

[B29] Bolos V Peinado H Perez-Moreno MA Frga M Esteller M Cano A The transcription factor Slug represses E-cadherin expression and induces epithelial to mesenchymal transitions: a comparison with Snail and E47 repressors J Cell Sci 2002 116 499 511 12508111 10.1242/jcs.00224

[B30] Cicchini C Filippini D Coen S Marchetti A Cavallari C Laudadio I Spagnoli FM Alonzi T Tripodi M Snail controls differentiation of hepatocytes by repressing HNF4alpha expression J Cell Physiol 2006 209 230 238 10.1002/jcp.20730 16826572

[B31] Tanaka T Jiang S Hotta H Takano K Iwanari H Sumi K Daigo K Ohashi R Sugai M Ikegame C Umezu H Hirayama Y Midorikawa Y Hippo Y Watanabe A Uchiyama Y Hasegawa G Reid P Aburatani H Hamakubo T Sakai J Naito M Kodama T Dysregulated expression of P1 and P2 promoter-driven hepatocyte nuclear factor-4alpha in the pathogenesis of human cancer J Pathol 2006 208 662 672 10.1002/path.1928 16400631

[B32] Ning BF Ding J Yin C Zhong W Wu K Zeng X Yang W Chen YX Zhang JP Zhang X Wang HY Xie WF Hepatocyte nuclear factor 4 alpha suppresses the development of hepatocellular carcinoma Cancer Res 2010 70 7640 7651 10.1158/0008-5472.CAN-10-0824 20876809

[B33] Lazarevich NL Cheremnova OA Varga EV Ovchinnikov DA Kudrjavtseva EI Morozova OV Fleishman DI Engelhardt NV Duncan SA Progression of HCC in mice is associated with a downregulation in the expression of hepatocyte nuclear factors Hepatology 2004 39 1038 1047 10.1002/hep.20155 15057908

[B34] Fidler IJ Poste G The “seed and soil” hypothesis revisited Lancet Oncol 2008 9 808 10.1016/S1470-2045(08)70201-8 18672217

