# Supplementary material for: The role of hepatocyte nuclear factor 4alpha in metastatic tumor formation of hepatocellular carcinoma and its close relationship with the mesenchymal–epithelial transition markers
Source: BMC Cancer. 2013 Sep 23;13:432. doi: 10.1186/1471-2407-13-432 (PMC3852538; doi:10.1186/1471-2407-13-432)
Supplement: Additional file 1: Table S1 — Correlation between HNF4alpha, Snail, Slug, E-cadherin, Fibronectin, N-cadherin and Vimentin in primary tumors and their corresponding metastases samples. [file 1471-2407-13-432-S1.doc]

**Table S1 Correlation between HNF4alpha, Snail, Slug, E-cadherin, Fibronectin, N-cadherin and Vimentin in primary tumors and their corresponding metastases samples**

|  | | E-cadherin | Fibronectin | N-cadherin | Vimentin |
| --- | --- | --- | --- | --- | --- |
| Snail | Pearson Correlation | -.444(**) | .373(**) | .518(**) | .391(**) |
|  | Sig. (2-tailed) | .000 | .003 | .000 | .002 |
|  | N | 62 | 62 | 62 | 62 |
| Slug | Pearson Correlation | -.481(**) | .426(**) | .282(*) | .294(*) |
|  | Sig. (2-tailed) | .000 | .001 | .026 | .021 |
|  | N | 62 | 62 | 62 | 62 |
| HNF4alpha | Pearson Correlation | .730(**) | -.382(**) | -.190 | -.228 |
|  | Sig. (2-tailed) | .000 | .002 | .138 | .075 |
|  | N | 62 | 62 | 62 | 62 |

** Correlation is significant at the 0.01 level (2-tailed).

* Correlation is significant at the 0.05 level (2-tailed).
